# Homeolog expression analysis in an allotriploid non-model crop via integration of transcriptomics and proteomics

**DOI:** 10.1038/s41598-018-19684-5

**Published:** 2018-01-22

**Authors:** Jelle van Wesemael, Yann Hueber, Ewaut Kissel, Nádia Campos, Rony Swennen, Sebastien Carpentier

**Affiliations:** 1 Laboratory of Tropical Crop Improvement, KU Leuven, Willem Decroylaan 42, Leuven, Belgium; 2 Bioversity International, Parc Scientifique Argropolis II, Montpellier, France; 3 Bioversity International, Willem Decroylaan 42, Leuven, Belgium; 4 International Institute for Tropical Agriculture, C/O Nelson Mandela Institute of Science and technology, P.O. Box 44, Arusha, Tanzania; 5 Facility for SYstems BIOlogy based MAss spectrometry, Herestraat 49, Leuven, Belgium

## Abstract

The fate of doubled genes, from allopolyploid or autopolyploid origin, is controlled at multiple levels, resulting in the modern day cultivars. We studied the root growth of 3 different triploid banana cultivars under control and osmotic stress conditions. The root growth of the allopolyploid ABB cultivar was 42% higher under control and 61% higher under osmotic stress. By integrating transcriptomics and proteomics, we studied the gene expression of all 3 cultivars, resulting in 2,749 identified root proteins. 383 gene loci displayed genotype specific differential expression whereof 252 showed at least one Single Amino Acid Polymorphism (SAAP). In the ABB cultivar, allele expressions supposedly follow a 1/3 and 2/3 pattern for respectively the A and the B allele. Using transcriptome read alignment to assess the homeoallelic contribution we found that 63% of the allele specific genes deviated from this expectation. 32 gene loci even did not express the A allele. The identified ABB allele- specific proteins correlate well with the observed growth phenotype as they are enriched in energy related functions such as ATP metabolic processes, nicotinamide nucleotide metabolic processes, and glycolysis.

## Introduction

Bananas and plantains (*Musa* spp.) are a major allopolyploid crop with a yearly production of ±145 million tonnes (2014, FAOstat), spread over the (sub-)humid tropics, an estimated 85% of which coming from smallholder plots in the developing world^1^. Banana demands a lot of water and drought stress is the major limiting abiotic stress factor^2^. For example in the East-African highlands, where the crop evapotranspiration is between 1200 and 1300 mm per year, there is an 8–10% yield decline per 100 mm water that could not be transpired^2^. In the 21^st^ century, agriculture is challenged to feed an increasing population while minimizing unsustainable usage of (natural) resources. This means that crops (including banana as a top ten staple food crop) need to be better adapted to a changing and increasingly variable environment^3–6^. Fitness, survival, and agricultural productivity result from the plant genotype, environment interaction and Management (G × E × M). Flexibility of plants towards the environment is naturally determined by genetic diversity (G), and a deeper understanding thereof towards the phenotype is a priority^7,8^. It is a major objective for crop scientists to identify sources of natural variation with potential to rise the tolerance towards unfavourable (a)biotic constraints while minimizing the yield penalty^8^. The majority of plant research has focused on model species (*Arabidopsis thaliana*) and major, sequenced crops (e.g. *Oryza sativa*, *Zea mays, Glycine max*). However, many other important food, feed, or energy crops and their wild relatives are highly relevant in the quest for sustainable cultivars. Among these are numerous polyploid crops with complex heterologous genomes.

Modern *Musa* cultivars are triploid, hybrid crosses characterized by high levels of gene flow between members of the Musaceae family, mainly: *Musa acuminata* (A genome) and *Musa balbisiana* (B genome)^9,10^. The modern, seedless, vegetative clones originate from initial sexual reproductions between these seedbearing progenitor species^11,12^. Different levels of ancestor (A or B) genome contribution are noted. After the polyploidization, and consequent sterilization, genetic diversity was only enhanced by mutation^9^. Reference sequences have been published for the *Musa acuminata* (A) genome, and a draft reference sequence for the *Musa balbisiana* (B) genome^13–15^. Efforts have also been made to sequence crop wild relative species of banana (*Musa itinerans*) since these may be informative for modern banana cultivars^16^. The dissimilarity between the *Musa acuminata* and the draft *Musa balbisiana* reference genomes is 1 SNP per 39 bp^13^.

Polyploid crops such as banana are characterized by a broad genetic diversity, acquired by genome merging and doubling^17^. Autopolyploids are constituted by chromosome sets of closely related populations, while allopolyploids result from interspecific hybridization^18,19^. The genome redundancy provides means for genetic novelty and adaptability towards environmental cues^20^. Gene loss or silencing, neo- and/or sub-functionalization, intergenomic transfer, allele dominance/co-dominance, differences in transcription/translation efficiency and post translational modifications, exemplify how the genome, transcriptome, and proteome are regulated following polyploidization events^18–20^. The allo-/and autopolyploid genome is thus a patchwork of gene variants enabling many genotype × environment interactions. Features unique for a preferential phenotype at the gene variant level are most probable candidate markers controlling the observed phenotype.

Advances in next- generation sequencing techniques enable high- resolution linkage studies between gene variants and traits of interest (Genome- Wide Association Studies, GWAS). However, the high degree of heterozygosity and the amount of replicated sequence reads in polyploid crops is a great challenge for effective single nucleotide polymorphism (SNP) calling^8,21^. In banana GWAS has been successfully applied in diploids for the seedless phenotype, a trait underpinned by a limited number of genes^12^. But as stated, this approach on the allopolyploid triploids for a complex multi gene trait is cumbersome. The link between gene or protein abundance and SNP or single amino acid polymorphisms (SAAP) and a preferential phenotype is more stringent with knowledge of the tissue, time point and environmental condition of the gene/protein activity, i.e. using functional genomics (transcriptomics and/or proteomics)^22^. RNA sequencing effectively combines gene expression quantification with gene sequencing and allows SNP calling. Yet, most current day read mapping software is written for diploid species and have difficulties to process complex (polyploid) genomes. The read mapping efficiency to the reference genome might be biased, and the degree of heterozygosity greatly increases computational effort, hampering quantitative results per genome^23^. Consequently the RNA reads are not separated and traced back to their (sub-) genome. Algorithms like PolyCat and HANDS2 process reads based on classification towards their genome of origin, but heavily depend on the presence and the quality of reference genomes^23,24^. Since not all cultivars of interest carry the reference sequence, mapping efficiency biases can still occur when one reference genome is more closely linked to a constituting genome than the other^23^.

Proteomics is a bottom-up, specific technique allowing to pick up and quantify the actual differential product as it is controlling the phenotype. Gel- based proteomics is very useful to identify allele- specific protein isoforms in a non-model crop^25^. This is exemplified in the HSP70 protein family in banana^26^. But, for multigenic traits, like tolerance to abiotic stress, it is important to get a broader picture. Via LC-MSMS multiple allele- specific products (tryptic specific peptides) are quantified without required prior knowledge^27^. Peptide identification is greatly enhanced with the availability of a reference genome. In reality, in non-model crops the database of the nearest related taxon can also be used. In essence LC-MSMS is suitable for the study of specific allelic contributions towards a preferential phenotype in polyploid non-model crops, eg. Hu *et al*.^28^ discovered 558 allele- specific protein isoforms in *Gossypium* using iTRAQ.

This study aims to select alleles that are correlated to drought stress in banana (Musa spp.), an allopolyploid, non-model crop. Therefore we rely on a lab model mimicking drought stress using an osmotic stress agent (Poly-Ethylene Glycol, PEG-8000). Since different protein isoform(s)/transcript(s)/gene copy(s) are controlling the phenotype, it is crucial to identify genes specific to the favorable phenotype. The ensemble of genes, controlled by their own promotors with their own properties, steer the phenotype. The identified specific alleles can help to unravel the genetic basis attributing to drought tolerance in banana. Hence a workflow to study quantitatively the gene variants is indispensable. Allele- specific gene responses are picked up in this workflow as (1) non-identical isoforms matching the same gene locus, and (2) as products that are higher expressed in one cultivar than in others.

## Results

### Assessment of root biomass under control and osmotic stress

**Figure 1:**
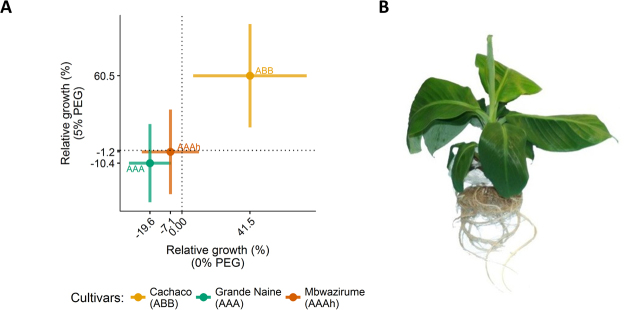
Relative root growth of AAA, AAAh, and ABB cultivars after 21 days of treatment (0 or 5% PEG. (**A**) Normalized dry weight of roots calculated by subtracting a plants dry weight from the treatment group median. Medians per cultivar are indicated with Inter Quartile Range. (**B**) Banana plant grown in the experimental setup.

The growth of three different genotypes under control and osmotic stress (5% PEG) conditions is observed after 21 days (Supplementary Fig. S1). Cachaco showed a relative growth 41.5% and 60.5% higher than the median of all control and stressed plants, respectively (Fig. 1). Cachaco has a significantly higher root growth than Grande Naine and Mbwazirume under 0% and 5% PEG conditions (Tukey HSD test, α = 0.05).

### The root proteome

**Figure 2:**
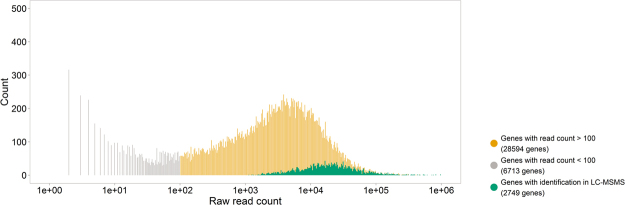
Identification of LC-MSMS spectra is correlated with the mRNA read count. Histogram with total read count in 18 samples visualized. Yellow: all genes in quantitative mRNA, green: all genes with identification in LC-MSMS, grey: genes with read counts below 100.

To unravel the molecular mechanisms behind the observed phenotypes, 234,739 features were aligned and quantified, 70,899 were targeted for MS2 (Supplementary Fig. S2). 51,765 spectrum sequence matches were found by integration of three search engines (Mascot, x!Tandem and MS-GF+) searching cultivar specific databases. In total 5,261 peptide sequences were successfully identified in 2,749 unique proteins. The majority of successful, peptide sequence identifications was based on results from all three search engines (4,143), 423 identifications were unique for the Mascot search engine, and 243 and 46 for x!Tandem and MS-GF+, respectively (Supplementary Fig. S3). The identified proteins correspond to transcripts (mRNA) with relatively high abundance (Fig. 2). The peptides taken up in MS2, are generally found more in higher abundant spectra. MS1 peptides span 6 orders of magnitude 10, those targeted for MS2 3 orders, and the identified peptide sequences approximately 2 orders of magnitude (Supplementary Fig. S2).

### Integration of proteomics with transcriptomics calls cultivar specific alleles

**Figure 3:**
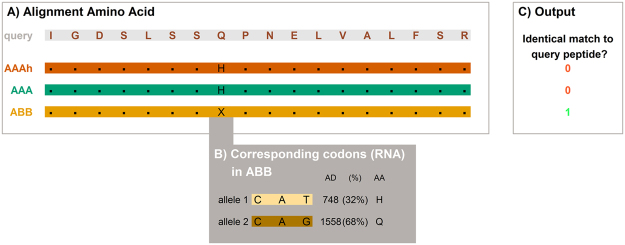
Integration of transcriptomics with proteomics to select cultivar specific alleles: assessment of aligned amino acid sequences and codons (RNA) reveals polymorphisms underlying the specific peptides. Exemplified for Ma02_t23730 (Sucrose Synthase 2). (**A**) Query peptide sequence identified in LC-MSMS (IGDSLSSQPNELVALFSR) aligned to AAA, AAAh, and ABB databases. (**B**) For the ambiguous amino acid in ABB database (X) the variants of the corresponding codon are assessed in the RNA database of the cultivar of interest. The allele depth (AD) of 1558 (68%) for allele 2 favors the presence of a unique amino acid (AA). (**C**) This is translated into specificity matching output.

A locus (plural loci) in genetics is the position of a gene on a chromosome. The 5,261 validated feature sequences are spread over 2,749 loci (Supplementary Tables S1 and S2). A variant of the similar DNA sequence located at a given locus is called an allele. To call cultivar specific allelic sequences, all identified peptides were BLASTp searched across the 3 cultivar specific databases and the best match for each database was retained (Supplementary Table S2). The specificity of alignment is compared over the three cultivar specific databases. The workflow is exemplified for a query peptide (IGDSLSSQPNELVALFSR) identified in Ma02_t23730 (Sucrose Synthase 2; Fig. 3). A match on the same locus with less than 100% identity in another database identifies a cultivar specific peptide (Fig. 3A, Supplementary Fig. S4). The possibilities underlying the ambiguous amino acid X, were assessed at the transcriptome level (2 possible codons) at the putative SAAP location (Fig. 3B, Supplementary Fig. S4). The specific read count for the codons revealed whether or not the query amino acid was to be found at this locus. In this example case the query could be found, as such an ABB specific peptide sequence was identified (Fig. 3C).

Following this workflow (Supplementary Fig. S4), the integration of proteomics with transcriptomic validation identifies 252 loci with allelic isoforms. This is exemplified for 2 genes annotated as involved in glycolytic processes in Table 1, and can be found in Supplementary Table S3 for all the allele- specific proteins.

**Table 1:** Subset of the identified specific loci in 2 genes involved in glycolytic processes.

Gene	Locus	Function	Variety	Sequence	Mascot	MS-GF+	X!Tandem
Ma02_t23730	chr02:28633167-28639039	sucrose synthase 2-like	Cach	IGDSLSSQPNELVALFSR	92.11	noMatch	noMatch
Ma02_t23730	chr02:28633167-28639039	sucrose synthase 2-like	GN	IGDSLSSHPNELVALFSR	20.63	1.66E-12	0.00044
Ma02_t23730	chr02:28633167-28639039	sucrose synthase 2-like	Mbw	IGDSLSSHPNELVALFSR	20.63	1.66E-12	0.00044
Ma08_t33800	chr08:44000904-44004899	phosphoglycerate kinase, cytosolic-like	Cach	VDLNVPLDDNQKITDDTR	40.79	noMatch	noMatch
Ma08_t33800	chr08:44000904-44004899	phosphoglycerate kinase, cytosolic-like	GN	VDLNVPLDDNLKITDDTR	54.55	2.34E-11	0.021
Ma08_t33800	chr08:44000904-44004899	phosphoglycerate kinase, cytosolic-like	Mbw	VDLNVPLDDNLKITDDTR	54.55	2.34E-11	0.021

The alternative cultivar specific sequences are provided. Identification scores from Mascot, msgf and x!Tandem search algorithms are given, noMatch: peptide not identified through this search algorithm.

**Figure 4:**
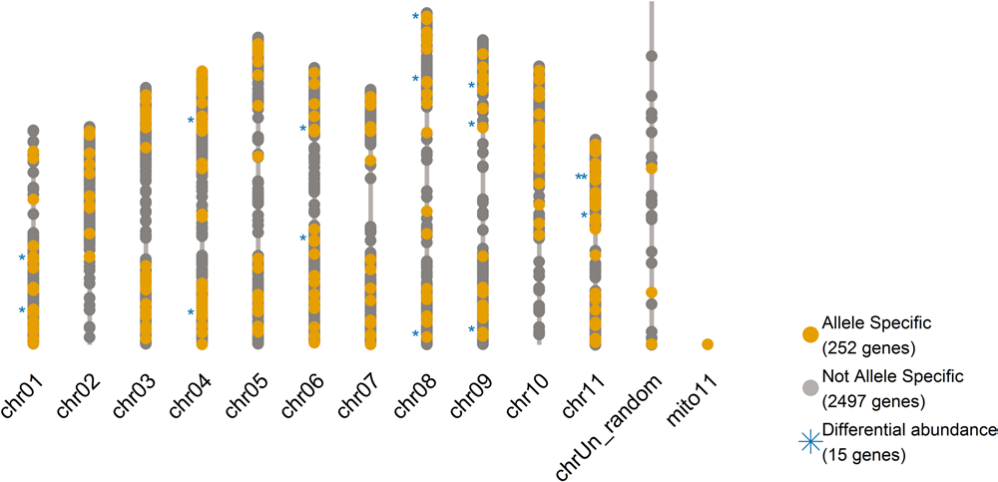
All identified gene loci, indicated when evidence (SAAP) for cultivar specificity was found. Differential peptide abundance between control (0% PEG) and osmotic stress (5% PEG) was assessed using the Kruskal Wallis test (α = 0.1) in the ABB and AAA cultivars using 3 or 6 biological replicates respectively.

Figure 4 presents an overview of the loci identified with sequence specificity. 15 loci were linked to a differential abundance of the peptides in 0% PEG relative to 5% PEG samples (Kruskal-Wallis test, α = 0.1) (Fig. 4).

### The relative abundance of allelic isoforms in the tolerant polyploid phenotype

**Figure 5:**
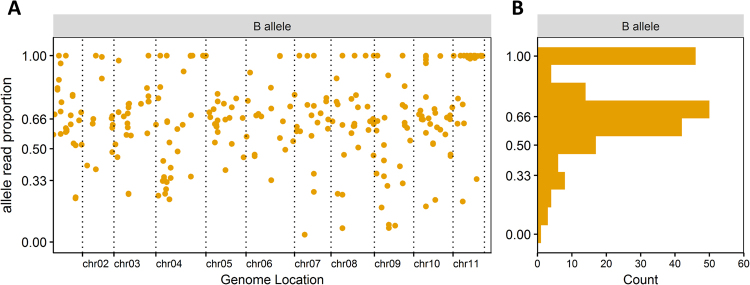
Allele- specific mRNA read proportions at SAAP locations (loci found in uniquely in ABB at the proteomic level) vary between 0 and 1 for the B alleles (**A**). Distribution pattern of the read proportion shows homeolog expression (bias) (**B**). mRNA reads assessed in ABB control samples, separated based on the RNA (codon) translation into ABB and non-ABB specific amino acid sequences.

The drought tolerant phenotype, Cachaco (ABB) carries two copies of the *Musa balbisiana* genome, and one of *Musa acuminata*^29^. Our workflow (Supplementary Fig. S4) identified both allele- specific protein sequences. The relative read distribution (mRNA) of these subgenome copies has been assessed and the read proportion is displayed as the ratio of B specific reads (Fig. 5A) over the total number of reads for the locus. The read proportion is distributed around 0.66 for the B alleles (Fig. 5B). 123 loci, dispersed all over the *Musa* genome, show intermediate read proportions not matching the expected 2/3^rd^ read distribution. In 32 loci, only one homeoallele was expressed (Supplementary Table S4).

### Quantitative allele- specific expression in contrasting phenotypes

**Figure 6:**
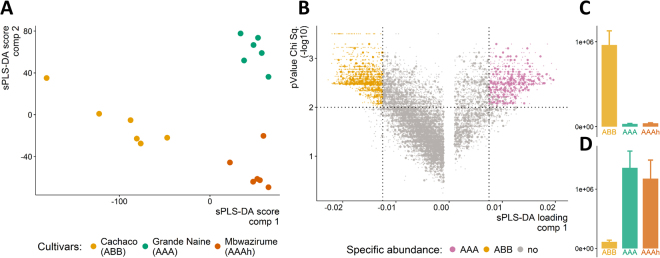
Quantitative selection of cultivar specific proteome features based on uni- and multivariate statistics. (**A**) Sparse partial least squares separates genomic groups (AAA and ABB) in Component 1. (**B**) Separation of features based on quantitative LC-MSMS. X-axis: sPLS-DA component 1: cut-off for selection <−0.0125 and >0.0075. Y-axis: −log10 of p-value of Chi Square Kruskal Wallis test with Benjamini-Hochberg p-value adjustment. P-value of 0.01 used as cutoff for Y axis. (**C**,**D**) Examples of ABB and AAA specific expression pattern respectively.

Based on the peptide intensity, a general quantitative overview of the root proteome displays the proteomic diversity between cultivars. Diversity between genomic subgroups controls the phenotypic divergence observed in Fig. 1. We are interested in mechanisms underlying the observed phenotypes. Sparse partial least squares discriminant analysis (sPLS-DA) discriminates genomic constitutions based on peptide abundance (ABB, AAAh, AAA) (Fig. 6A). Component 1 separates ABB from AAA samples, while component 2 separates Grande Naine and Mbwazirume, both AAA but originating from different subgroups of cultivated varieties: Cavendish and Mutika-Lugujira (East African Highland) bananas respectively. The result of sPLS-DA ranks different peptides in their specificity towards the cultivars. The combination of multivariate (sPLS-DA, horizontal axis, Fig. 6B) and univariate statistics (Kruskal Wallis, vertical axis; Fig. 6B) allows to identify peptides correlated with the drought tolerant phenotype (ABB specific, or AAA specific). Figure 6C and D show examples of the peptide abundance of AAA respectively ABB specific LC-MSMS features. This quantitative selection method identifies 131 additional genes with an ABB or AAA specific quantitative abundance profile, 29 of these are involved in glycolytic processes (Fig. 6, Table 2, see Supplementary Table S5 for all quantitatively selected specific proteins).

**Table 2:** Selection of mass identical proteome features with cultivar specific abundance.

Gene	Locus	Function	Sequence	Specificity	sPLS-DA comp.1 (a)	pChi (b)	Groups (c)
Ma07_t11500	chr07:8539813-8543320	Alcohol dehydrogenase-like 7	IIGVDLNPDKFEIGK	AAAspec	0.012196	0.00334	b-a-a
Ma05_t27700	chr05:38886905-38891995	glyceraldehyde-3-phosphate dehydrogenase 2, cytosolic	IVSNASCTTNCLAPLAK	ABBspec	−0.01322	0.003399	a-b-b
Ma09_t02110	chr09:1536831-1540788	glyceraldehyde-3-phosphate dehydrogenase 2, cytosolic-like	IVSNASCTTNCLAPLAK	ABBspec	−0.01322	0.003399	a-b-b
Ma08_t33830	chr08:44031272-44038116	glyceraldehyde-3-phosphate dehydrogenase GAPCP1, chloroplastic-like	IVSNASCTTNCLAPLAK	ABBspec	−0.01322	0.003399	a-b-b
Ma05_t00210	chr05:152861-158287	glyceraldehyde-3-phosphate dehydrogenase GAPCP2, chloroplastic-like	IVSNASCTTNCLAPLAK	ABBspec	−0.01322	0.003399	a-b-b
Ma03_t32880	chr03:34484915-34490528	hexokinase-2-like	SDELFDFIASALVK	AAAspec	0.012206	0.002754	b-a-a
Ma04_t16550	chr04:15949921-15954721	malate dehydrogenase, mitochondrial	ALEGSDVVIIPAGVPR	ABBspec	−0.02077	0.00108	a-c-b
Ma04_t16550	chr04:15949921-15954721	malate dehydrogenase, mitochondrial	LNPLVSNLALYDIAGTPGVAADVGHINTR	ABBspec	−0.01539	0.002117	a-b-b
Ma05_t03680	chr05:2710087-2716309	malate dehydrogenase-like	AGEWTIVQGLSVDEFSR	ABBspec	−0.014	0.006136	a-b-b
Ma01_t19240	chr01:14718937-14723088	phosphoglycerate kinase, cytosolic, transcript variant X2	ELDYLVGAVANPK	ABBspec	−0.0131	0.002117	a-b-b
Ma05_t00310	chr05:217490-221221	phosphoglycerate kinase, cytosolic-like	ELDYLVGAVANPK	ABBspec	−0.0131	0.002117	a-b-b
Ma05_t00310	chr05:217490-221221	phosphoglycerate kinase, cytosolic-like	LISALPDGGVLLLENVR	ABBspec	−0.01825	0.002754	a-b-b
Ma11_t09970	chr11:9163536-9171836	Putative Pyruvate kinase, cytosolic isozyme	LGDLYQTQIFAK	ABBspec	−0.01351	0.003096	a-b-b
Ma06_t14950	chr06:10182092-10190553	Putative Pyruvate kinase, cytosolic isozyme	LGDLYQTQIFAK	ABBspec	−0.01351	0.003096	a-b-b
Ma00_t00620	chrUn_random:1791125-1796085	Pyruvate kinase, cytosolic isozyme	ANIDIDGILKELPNDGRVPK	ABBspec	−0.01532	0.002552	a-b-b
Ma05_t31060	chr05:41159306-41165776	triosephosphate isomerase, cytosolic	VASADVVDVVVSPPFVFLPLVK	ABBspec	−0.01924	0.003321	a-b-b

Example data for proteins involved in glycolytic processes are shown. Feature selection is based on sparse partial least squares discriminant analysis (sPLS-DA) component 1 (a), Kruskal Wallis p-value (b), indicated by groups (Cachaco - Grande Naine-Mbwazirume) (c), a > b > c.

### Functional annotation of alleles

252 loci were found containing a SAAP, and an additional 131 loci with a phenotype specific abundance profile, without SAAP, were picked up. To characterize alleles correlated with enhanced root growth, we performed a gene ontology (GO) enrichment analysis on all allele- specific proteins. Gene ontology enrichment of these 383 loci relative to the 2,749 identified root proteins is shown in Supplementary Table S6. Energy related functions such as ATP metabolic process, nicotinamide nucleotide metabolic process, glycolysis and tricarboxylic acid cycle are enriched and correlate well with the observed growth phenotype.

## Discussion

### Characterization of polyploid non-model crops

Numerous studies have been undertaken to study the phenotypic and genetic variation of crops in relation to drought tolerance^30–33^. In this study we observe a distinct phenotype: Cachaco (ABB) shows a significantly higher root dry mass compared to AAA cultivars, both under control and stress conditions (41.5 & 60.5% relative to the median plant per treatment respectively; Fig. 1). Enhanced root growth is a drought avoidance/postponement mechanism and water uptake is (temporarily) ensured when deeper water layers are reached^34^. Typically deeper soil layers are less influenced by a decline in soil hydraulic conductivity in a drying environment^35^. Water uptake is determined by the exploration of a particular soil layer by the root biomass, and therefore deeper rooting varieties are possibly more drought tolerant by avoiding drought^36^. A minimal setback of root growth under variable soil moisture contents stabilizes plant performance during (limited) stress periods^37,38^. The main aim of this study was to pick up allele- specificity correlated to the favourable phenotype at the protein level in a relevant tissue and treatment. This eventually leads to better understanding of the genetic variation in a drought tolerant phenotype.

### Integration of quantitative LC-MSMS proteomics with transcriptomics enables to call allele- specificity in a high-throughput way

Advanced whole genomic screening experiments enable the identification and interpretation of mutations at the genome level^39^. We developed a workflow to identify variants at the allele level, as our interest lies in the differential contributions of diverse subgenomes to the allopolyploid phenotype. Transcriptome as well as proteome- wide studies of these three contrasting phenotypes reveal differentiation between ABB and AAA transcripts and peptides in the first sPLS component whereas the differentiation between both AAA cultivars is taken up in the second component (Fig. 6)^30^. This characterizes the genetic diversity: within the three genotypes, both AAA resemble each other more closely than the ABB in these test conditions. These findings are convincing to study the differences between AAA and ABB in detail, at the allelic level. Polyploid crops are characterized by a multitude of gene variant × environment interactions. This heterogeneity makes quantitative, whole genome studies complex, but it is also the basis of the observed phenotypic differences. There is a need for workflows enabling the study of genetic heterogeneity, with respect to the unique alleles. At the same time allele- specificity calling provides an opportunity to look into the homeolog expression (bias).

Linkage studies between a preferential phenotype and nucleotide/amino acid polymorphisms identify genes putatively involved in the trait of interest. However, the sterile and polyploid nature of banana, where cultivars exist of many crossed inter specific hybrids, hamper the use of SNP mapping in association studies, especially with multi gene responses like abiotic stress tolerance^12^. Integration of proteomics with transcriptomics is a bottom-up approach, enabling allele- specificity calling, proteome- wide, in relevant tissues, time points and environmental conditions. The proteogenomics workflow elegantly proves the existence of 252 genome locations carrying polymorphisms between contrasting phenotypes. The combination of univariate and multivariate statistics on LC-MSMS abundances picks up allele- specific expression in another 131 genes, even without a causal SAAP at the genomic location (Fig. 6). Together they form (a subset of) the genetic basis for the phenotype of preference. Once validated in multiple cultivars the polymorphisms/locations are promising markers for selection for climate-smart cultivars, as they are evidenced at the protein level, and correlated to a more drought tolerant phenotype.

### The impact of a tripled genome

After an allopolyploidy event two or more diverse genomes hybridize in a single organism. This implies the phenotype of this organism is characterized by a mosaic of different gene responses inherent to these diverse constituting genomes^40,41^. The genomes are regulated at the genetic, transcriptomic, proteomic, and epigenetic level, which results in a multitude of possibilities for expression levels and thus the phenotype^42^. The tolerant cultivar of interest in our study, Cachaco, is constituted by two *Musa balbisiana* and one *Musa acuminata* genomes^29^. In banana, it has been suggested that the presence of the *Musa balbisiana* genome contributes to drought tolerance, thus the specific B isoforms and the relative abundance thereof are of special interest^25,32,43–46^.

Several low throughput studies reported that the allelic variants in banana are not necessarily equally expressed^25,26,41,46^. Alleles act in part independently and every homeolog can contribute equally, or not, to the final phenotype. Homeolog expression bias favors allelic isoform(s) over another^42^. This suggests differential regulation and efficiency of the involved proteins at the allelic level, driving expression levels of genes and genomes to a preferential phenotype^47^. In the ABB cultivar, we expect allelic copies of a gene to be transcribed in relative proportions of 1/3 and 2/3 for the constituting A and B alleles respectively. Nevertheless, positive and negative homeolog expression bias is observed at 123 loci (Fig. 5, Supplementary Table S4). Positive expression bias, loci where the relative read contribution for B alleles is higher than 0.66, identifies genes where the B isoform is prevalently expressed in this specific time point and in this specific tissue. It is unclear what mechanism underlies the homeolog expression bias and how stable it is.

An extreme case of homeolog expression bias is called expression level dominance: the total gene expression is dominated by the expression of one of the homeologs, while the alternative variant is not expressed^48,49^. Previously, in a low throughput study the absence of the encoding gene sequence of an A homeolog of the Abscisic Acid stress ripening Asr-gene was already noted in our ABB banana^46^. This proves that expression level dominance occurs in triploid bananas, possibly due to backcrossing events during the formation of triploid banana cultivars^41^. In our study, we found 32 genes that are characterized by expression level dominance: an allele- specific polymorphism unique to the ABB specific isoform (100% relative read contribution) (Fig. 5, Supplementary Table S4). For example Ma11_t17540 (Glyceraldehyde 3 phosphate dehydrogenase), Ma11_t00520 (Aquaporin PIP2-3), Ma08_t09170 (Polyphenol oxidase). The AAA specific copy in these 32 genes is not actively transcribed. Expression level dominance could be the result of sub-/neofunctionalization of genes, but might also be tissue specific or time point specific expression, addressing the subgenomes in a different manner^50^. It might be the result of epigenetics, chromosome rearrangements, gene loss,… causing the non-favored gene (region) to be silenced or dropped.

Homeolog expression bias offers great flexibility to polyploid crops as this is not a static feature under various abiotic stresses. For example, a number of genes in the allopolyploid *Coffea arabica* cv. Java showed similar expression patterns compared with the parental *C. canephora* under hot conditions, while no parental preference was exhibited under cool conditions^51^. In a synthetic allotetraploid *Arabidopsis*, the ratio between expression levels of homeologs of the same loci changed significantly under cold stress in 1.11% of the identified homeologs^52^. GO enrichment revealed that the majority of these genes was involved in cold stress responses. These cases of homeolog expression bias under various environmental conditions exemplify the plasticity of allopolyploids and their relevance for agriculture^53^. However, not always can the link between homeolog expression bias and the environment be made at the genome level^54^. Genetic regulation in polyploids is complex. In similar subgenomes, the variations between homeologs are minor, and regulatory factors, like transcription factors, other binding factors, and epigenetic status, act independently of the subgenome^52,54,55^. Thus increased homeolog expression bias can highlight the local dissimilarity between subgenomes, and the specificity of one of the subgenomes to the prevailing environment.

### ABB specific proteins are involved in drought tolerance related pathways

Functional genomic techniques (proteomics and transcriptomics) provide insights in a snapshot of the (metabolic) activities in root tissue under control and osmotic stress conditions at a specific time point. Under drought stress, the metabolic responses of roots and shoots are opposite: a relative investment in root metabolism coincides with a decreased growth metabolism in autotrophic shoots^37,56^. Roots are non-photosynthetic and depend entirely on respiration of assimilates synthesized aboveground. However, under (drought) stress conditions, an imbalance between photosynthesis and respiration occurs, hence energy efficiency at root level is interesting towards drought tolerance^30,57,58^.

In this study 383 gene loci were selected with sequence uniqueness or increased protein abundance specific for the cultivar displaying enhanced root growth. The functional annotation of these genes via gene ontology (GO) enrichment, shows that the identified allele- specific genes are a non-random subset of genes (Supplementary Table S6). There is a significant presence of proteins involved in pathways related to energy and metabolism, the glycolytic pathway for example (GO: 0006096, p-value 0.002). The *Musa* root response to osmotic stress is characterized by enhanced respiration, glycolysis and fermentation pathways^30^. Glycolysis is a major pathway which needs to produce reductants (NAD(P)H) and carbohydrates for anabolic metabolism. Within the glycolytic pathway, 36 genes with a SAAP or quantitative cultivar specificity are identified (Table 1, Supplementary Table S3).

The identified specific genes are involved in general abiotic stress tolerance related pathways. Under higher respiratory status, attributed to osmotic stress, increased ROS production is inevitable. The mitochondrial electron transport chain is a major site of ROS production^59,60^. Specific polymorphisms are found within several genes involved in the maintenance of cellular integrity under (ROS)-stress (Supplementary Table S3, S5). NADP-isocitrate dehydrogenase for example produces NADPH which helps to keep small antioxidants in reduced state so that ROS can be scavenged^59^. Heat Shock Proteins (HSP) are another class of genes involved in chaperoning enzymes, and maintenance of proteins in their correct state. With gel- based proteomics in banana, Vanhove *et al*.^26^ identified specific isoforms of HSP70 at 6 different genomic locations, one of which upregulated under osmotic stress. Two HSP genes (Ma02_t18000, Heat shock cognate 70 kDa protein, and Ma08_t21650, probable mediator of RNA polymerase II transcription subunit 37c) are corresponding between our study and Vanhove *et al*.^26^. Using LC-MSMS we identify 6 additional SAAP specific isoforms within the Heat Shock Protein (HSP) family and 4 additional HSP genes with cultivar specific abundance pattern. We demonstrate that the integration of proteomics with transcriptomics in a relevant tissue (root) at a relevant time point (3 days) in a relevant condition (osmotic stress) allows high throughput identification of genes with specific isoforms.

In conclusion, this study succeeded to select allele- specific protein variants by integration of proteomics and transcriptomics, leading to further understanding of the genetic diversity in an allopolyploid non-model crop. The allopolyploid gene expression is governed by a plethora of gene products, inherent to the multitude of gene copies. Our workflow discriminates the drought tolerant ABB from drought susceptible AAA cultivars at the allelic level. This finally allows homeolog specific quantification of gene expression, promising to make most out of (polyploid) breeding and biodiversity characterization/evaluation.

## Methods

### Plant material, growth conditions and osmotic stress treatment

Banana plants of three genotypes representing important cultivated subgroups were selected: Cachaco (Bluggoe subgroup; ABB; ITC0643), Grande Naine (Cavendish subgroup; AAA; ITC0180), and Mbwazirume (Mutika-Lujugira subgroup; AAA; ITC0084). Plantlets were obtained through the International *Musa* Transit Center (ITC, Bioversity International, hosted at KU Leuven, Belgium) Plants were grown in 500 mL PP containers in a plant incubator (Aralab Fitoclima Bio 600) where lights were set in a cycle of 12 h/12 h (light/dark), while the relative humidity and temperature were kept constant at 75% and 25 °C, respectively. Throughout plant growth the medium (305 mL) was renewed whenever the volume reached 55% of the original volume in at least one plant. The medium composition is: 361 mg/L KNO_3_, 121 mg/L K_2_SO_4_, 176 mg/L MgSO_4_. 7H2O, 181 mg/L MgCl_2_.6H_2_O, 194 mg/L KH_2_PO_4_, 398 mg/L NaH_2_PO_4_.2H_2_O, 464 mg/L Ca(NO_3_)_2_.4H_2_O, 105 mg/L CaCl_2_.2H_2_O, 60 mg/L Sequestrene, 1.1 mg/L H_3_BO_3_, 2.7 mg/L MnSO_4_.H_2_O, 0.23 mg/L ZnSO_4_.7H_2_O, 0.16 mg/L CuSO_4_.5H_2_O, 0.07 mg/L NaMoO_4_.2H_2_O, pH = 6 (modified from Swennen *et al*.^61^).

After 35 days of initial growth the stress treatment was initiated: the stressed subgroup received fresh medium including 5% of PEG-8000, an osmotic stress agent mimicking drought stress (pF 2.7). The control plants received a medium without addition of PEG-8000. 3 biological replicates per cultivar were harvested 3 days after the start of the 5% PEG-8000 treatment. Root tips for proteomic analysis (±4 cm long from the apex) were collected and the material was snap frozen in liquid nitrogen. 21 days after the start of the 5% PEG treatment, 6 biological replicates of each cultivar were phenotyped, measuring fresh and dry weight (dried 14 days, at 80 °C) of the roots.

### Protein extraction and data acquisition through mass spectrometry

Proteins were extracted following the phenol extraction/ammonium acetate precipitation protocol^62,63^. The digested samples (1 µg/5 µL) were injected and separated on an Ultimate 3000 UPLC system (Dionex, Thermo Scientific, USA) equipped with a Acclaim PepMap100 pre column (C18 3 µm-100 Å, Thermo Scientific, USA) and an C18 PepMap RSLC (2 µm, 50 × 50 cm, Thermo Scientific, USA) following a multiple step gradient with a linear gradient (flow rate: 0.300 µL/min): 4% buffer B (80% acetonitrile (CAN), in 0.08% formic acid (FA)) for 3 min, 4–12% in 17 min, 12–15% in 10 min, 15–18% in 45 min, 18–20% in 40 min, 20–30% in 65 min, 30–35% in 30 min, 35–65% in 10 min, 65–95% in 5 min, 95% for 40 min, 95–4% in 1 min, 4% for 39 min. The Q Exactive Orbitrap mass spectrometer (Thermo Scientific, USA) was operated in positive ion mode with a nano spray voltage of 1.5 kV and the source temperature set to 250 °C. ProteoMAss LTQ/FT-Hybrid ESI Pos. Mode Cal Mix (MSCAL5-1EA SUPELCO, Sigma Aldrich) was used as an external calibrant and the lock mass 445.12003 (polysiloxane) as an internal calibrant. The instrument was operated in data-dependent acquisition (DDA) mode with a survey MS scan at a resolution of 70,000 (fw hm at m/z 200) for the mass range of m/z 400-1,600 for precursor ions, followed by MS/MS scans of the top ten most intense peaks with +2, +3, and +4 charged ions above a threshold ion count of 1e^+^6 at 17,500 resolution using normalized collision energy (NCE) of 25 eV with an isolation window of 3.0 m/z, a dynamic exclusion of 30 s, and an Apex Trigger of 5–15 s. All data were acquired with Xcalibur 2.2 software (Thermo Scientific, USA). For identification and alignment, all raw data were converted into mgf-files using Progenesis (v 4.1, Nonlinear Dynamics, UK).

### Database construction and protein identification

Three cultivar specific databases were constructed based on mRNA seq reads from our plants (available at https://www.ncbi.nlm.nih.gov/bioproject/PRJNA305241,30). This transcriptomic dataset was realigned and recounted to the updated reference genome^14^. RNA-seq reads, contained in FASTQ files, were first trimmed based on low-quality ends and adapters were removed with cutadapt, v 2.7.9^64^. Quality was checked with FastQC (v0.11.3). Reads were then mapped against the *Musa acuminata* genome of reference (DH Pahang v2; http://banana-genome-hub.southgreen.fr/)^14,65^; using the splice junction mapper for RNA-seq STAR, v 2.5.0^66^ with default parameters. Read groups were added for each alignment, reads were split using SplitNCigarReads and locally realigned with InderlRealigner, v 3.4 (Genome Analysis ToolKit^67^) resulting in sample specific Binary Alignment/Map (BAM) files. Finally cultivar specific databases were constructed by concatenating BAM files per cultivar (mpileup, SAMTools^68,69^), followed by variant calling, in reference to the *Musa* reference genome v2 (45,855 sequences) (bcftools, SAMTools)^68,69^. Each database was complemented by 116 common repository of adventitious proteins (CRAP, http://www.thegpm.org/crap/) and combined into a target/decoy database^70^ enabling False Discovery Rate (FDR) estimation. The decoy sequences were created by reversing the target sequences in SearchGUI v 3.1.4^71,72^.

For every sample, peak lists (.mgf format) containing all MSMS spectra were extracted from Progenesis and were identified against its proper database integrating 3 database search algorithms: Mascot, v 2.2.06^73^, X!Tandem, version X!Tandem Vengeance (2015.12.15.2)^74^, and MS-GF+, v 10282^75^. The SearchGUI identification settings were as follows: enzyme trypsin, 2 missed cleavages (maximally), 10.0 ppm as MS1 and 0.02 Da as MS2 tolerance, Carbamidomethylation of C (+57.021464 Da) as fixed modification, Oxidation of M (+15.994915 Da), Acetylation of protein N-term (+42.010565 Da), Pyrolidone from E (−18.010565 Da), Pyrolidone from Q (−17.026549 Da), and Pyrolidone from carbamidomethylated C (−17.026549 Da) as variable modifications. The search was not conducted with error tolerance.

Peptides and proteins were inferred from the spectrum identification results using PeptideShaker v 1.14.5^71^. Peptide Spectrum Matches (PSMs), peptides, and proteins were validated at a 1.0% False Discovery Rate (FDR) estimated using the decoy hit distribution. Results were exported and processed further in R software (v 3.3.0) where PSMs were filtered, keeping the most confident peptide sequence matches. Only peptides identified in genes with average summed cultivar RNA seq read count above 100 were kept as trustworthy identifications. The mass spectrometry proteomics data have been deposited to the ProteomeXchange Consortium via the PRIDE partner repository with the dataset identifier PXD006375 and 10.6019/PXD006375^76^.

### Cross database allele- specific SAAP detection, validation, and quantification

To further validate the identified peptides, all peptides were searched across the 3 cultivar specific databases using the BLASTp algorithm^77^ and the best match for each database was retained. This approach is performed at the locus level, implying that peptides matching multiple loci are evaluated separately. Allele- specific variants are identified as peptides matching exactly (100%) in one database, while not in another database. In case of ambiguous proteins, containing amino acids depicted as X in the database, and caused by SNPs, inherent to the triploid banana nature, the underlying, possible mRNA variants were evaluated at this genome location. In these cases the uniqueness of sequence matching was evaluated by assessing the variant specific mRNA reads. ABB specific sequences, supposed to be the B alleles, are thus identified by their absence in AAA cultivars.

Quantifying homeoallele- specific reads was performed using Variant Call Format (VCF) files. These allowed to attribute the allele- specific read count to the identified allele- specific amino acid sequences in the control (0% PEG) samples of the allopolyploid Cachaco (ABB). The VCF files are developed using the mRNA seq SNPs, and are called by UnifiedGenotyper (GATK), with the ploidy parameter set to 3. Only SNPs with a mapping quality superior to 30 were kept and SNP clusters (more than 2 SNPs in 10 bp) were discarded. This was bundled into a VCF-file containing all biological replicates. Based on proteomic sequence information the allele- specific read count was assigned to its coding variant sequence. For every validated ABB specific allele variant we tested if the observed read ratio corresponds to what is expected based on the genomic constitution. The ABB cultivar, Cachaco, carries 1 copy of the *Musa acuminata* subgenome, and 2 copies of the *Musa balbisiana* subgenome^29^, hence it is expected that the A and B variants are expressed in a 1/3^rd^–2/3^rd^ ratio. This was tested by binomial testing using the number of ABB specific reads as number of successes, compared to the total number of reads for the specific locus, this with an expected ratio of 0.66. P-value adjustment was according to Benjamini-Hochberg.

### Multi- and univariate peptide statistics

Quantitative LC-MSMS was based on the peptide intensity using Progenesis. All samples were evaluated by Sparse Partial Least Squares Discriminant Analysis (sPLS-DA, mixOmics, R-package^78^), using genomic constitution groups (ABB, AAAh, AAA) as response variables. This multivariate statistical technique was complemented by the univariate, non-parametric Kruskal Wallis test (α < 0.01, p-value adjustment according to Benjamini-Hochberg; Agricolae, R-package).

### Functional Gene Ontology enrichment

*Musa* gene annotations were taken over from the Universal Protein Resource (UniProt). Our gene identifiers were converted to UniProt standard format (http://banana-genome-hub.southgreen.fr/convert; http://www.uniprot.org/uploadlists/) before Gene Ontology (GO) enrichment. We developed an in-house script to perform GO enrichment based on a user defined subset of genes in the UniProt format (https://labtrop.shinyapps.io/eRgo/). The tool is based on TopGO, R-package. Each molecular function (MF), biological process (BP) or cellular component (CC) is covered by a unique identifier, the GO ID. Data visualization as well as export of enriched GO terms are enabled in the tool. GO enrichment of all allele- specific proteins was performed against all 2,749 identified protein in the root proteome. We extracted all GO terms of biological process (BP) with significance level p < 0.05 (Fisher exact test).

## Electronic supplementary material

Supplementary figures

Table S1

Table S2

Table S3

Table S4

Table S5

Table S6
