# Supplementary material for: Homeolog expression analysis in an allotriploid non-model crop via integration of transcriptomics and proteomics
Source: Sci Rep. 2018 Jan 22;8:1353. doi: 10.1038/s41598-018-19684-5 (PMC5777989; doi:10.1038/s41598-018-19684-5)
Supplement: Supplementary file 1 — Supplementary figures [file 41598_2018_19684_MOESM1_ESM.pdf]

"Homeolog expression analysis in an allotriploid non-model crop via integration of transcriptomics and proteomics."

Jelle van Wesemael[1], Yann Hueber[2], Ewaut Kissel[1], Nádia Campos[1], Rony Swennen[1,3,4], Sebastien Carpentier[1,3,5], *

[1]Laboratory of Tropical Crop Improvement, KU Leuven, Willem Decroylaan 42, Leuven, Belgium.
[2]Bioversity International, Parc Scientifique Argropolis II, Montpellier, France
[3]Bioversity International, Willem Decroylaan 42, Leuven, Belgium
[4]International Institute for Tropical Agriculture, C/O Nelson Mandela Institute of Science and technology, P.O. Box 44, Arusha, Tanzania
[5]Facility for SYstems BIOlogy based MAss spectrometry, Herestraat 49, Leuven, Belgium
*Corresponding author: sebastien.carpentier@kuleuven.be

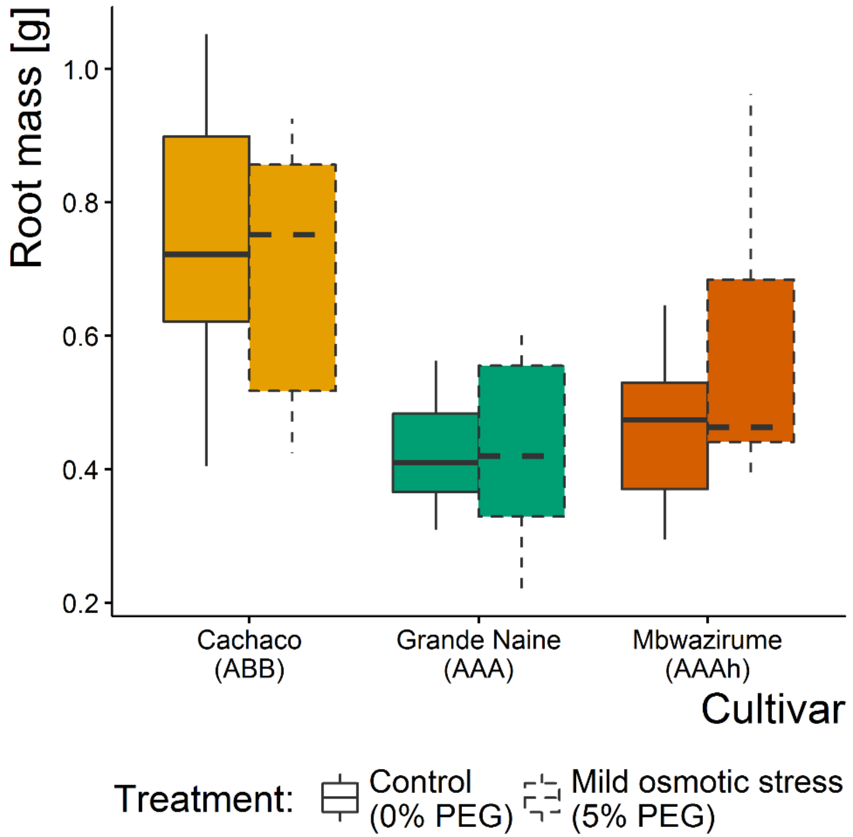

Supplementary Figure S1: Root growth of AAA, AAAh, and ABB cultivars after 21 days of treatment (0 or 5 % PEG).

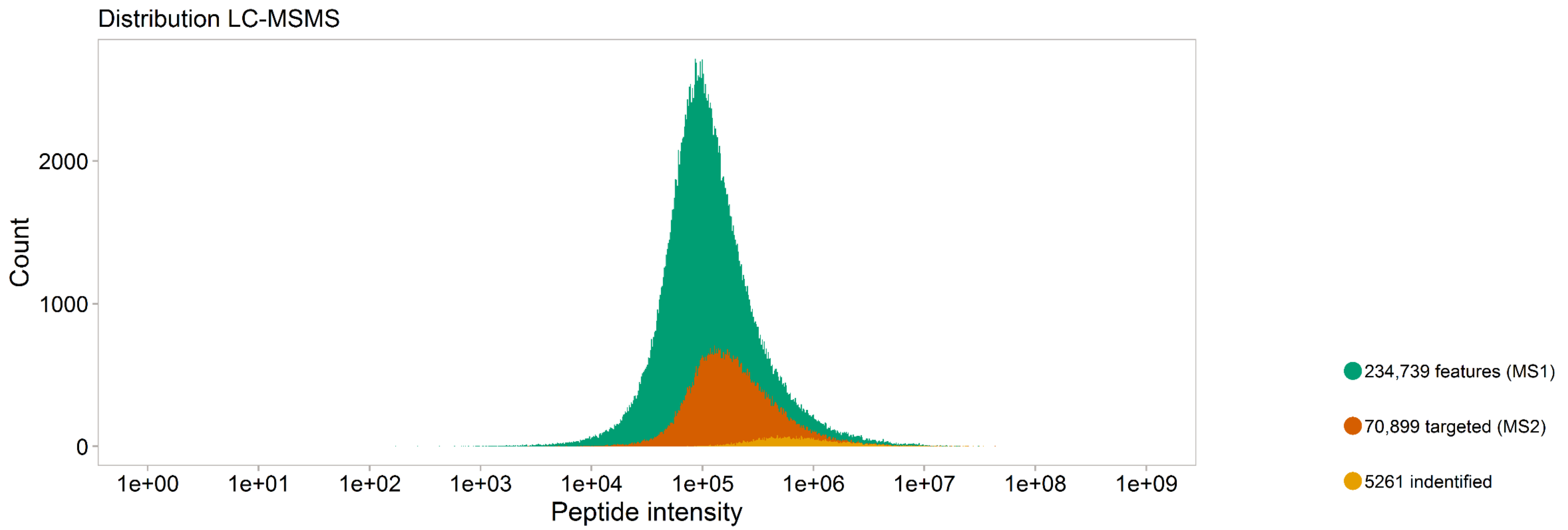

Supplementary Figure S2: Histogram of intensity of proteomic features taken along in MS1 and MS2 and leading to identification. Representation as histogram: x-axis: the peak intensity, y-axis: count. Green: only MS1, red: also MS2, yellow: peaks with identification.

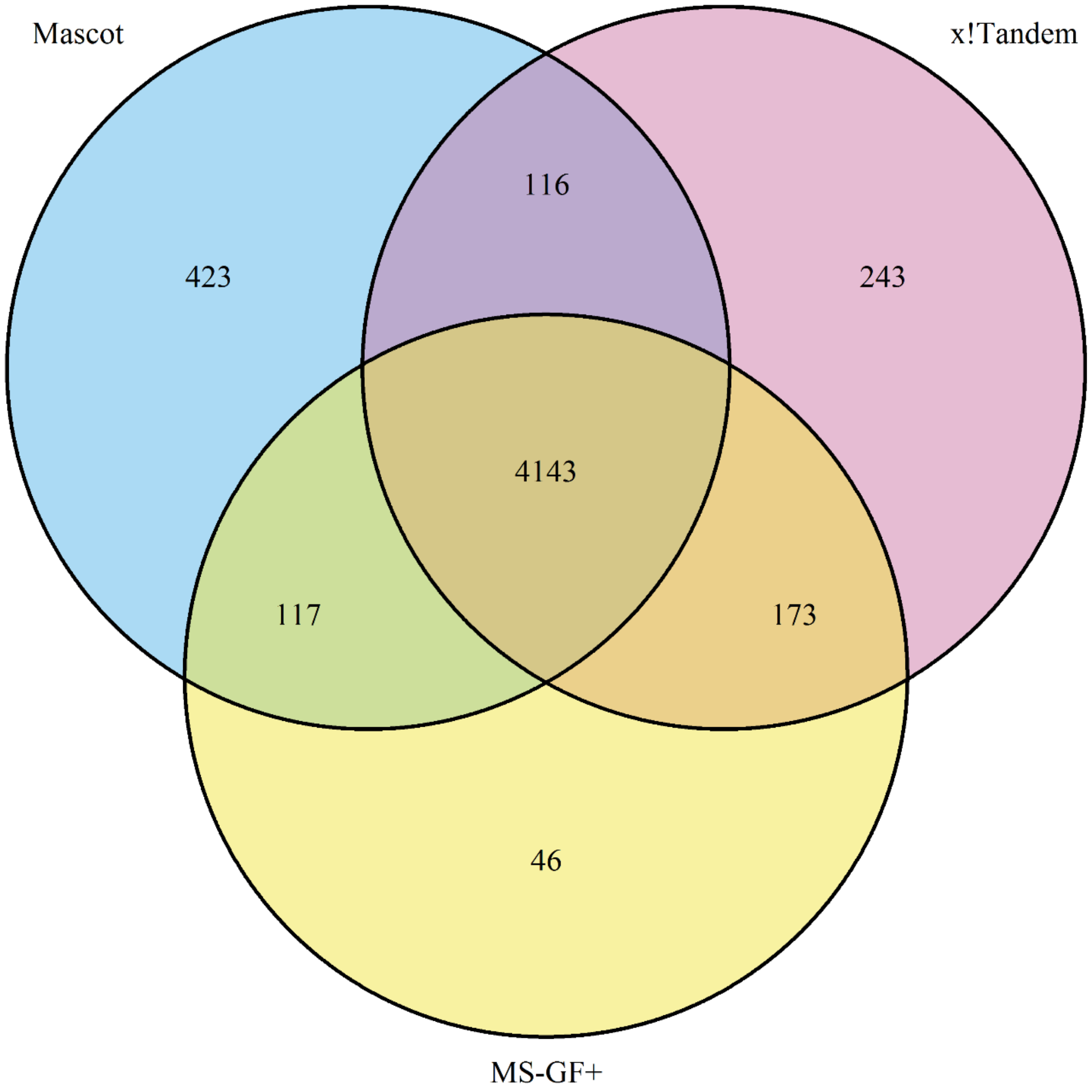

Supplementary Figure S3: The peptide identifications belonging to different search engines.

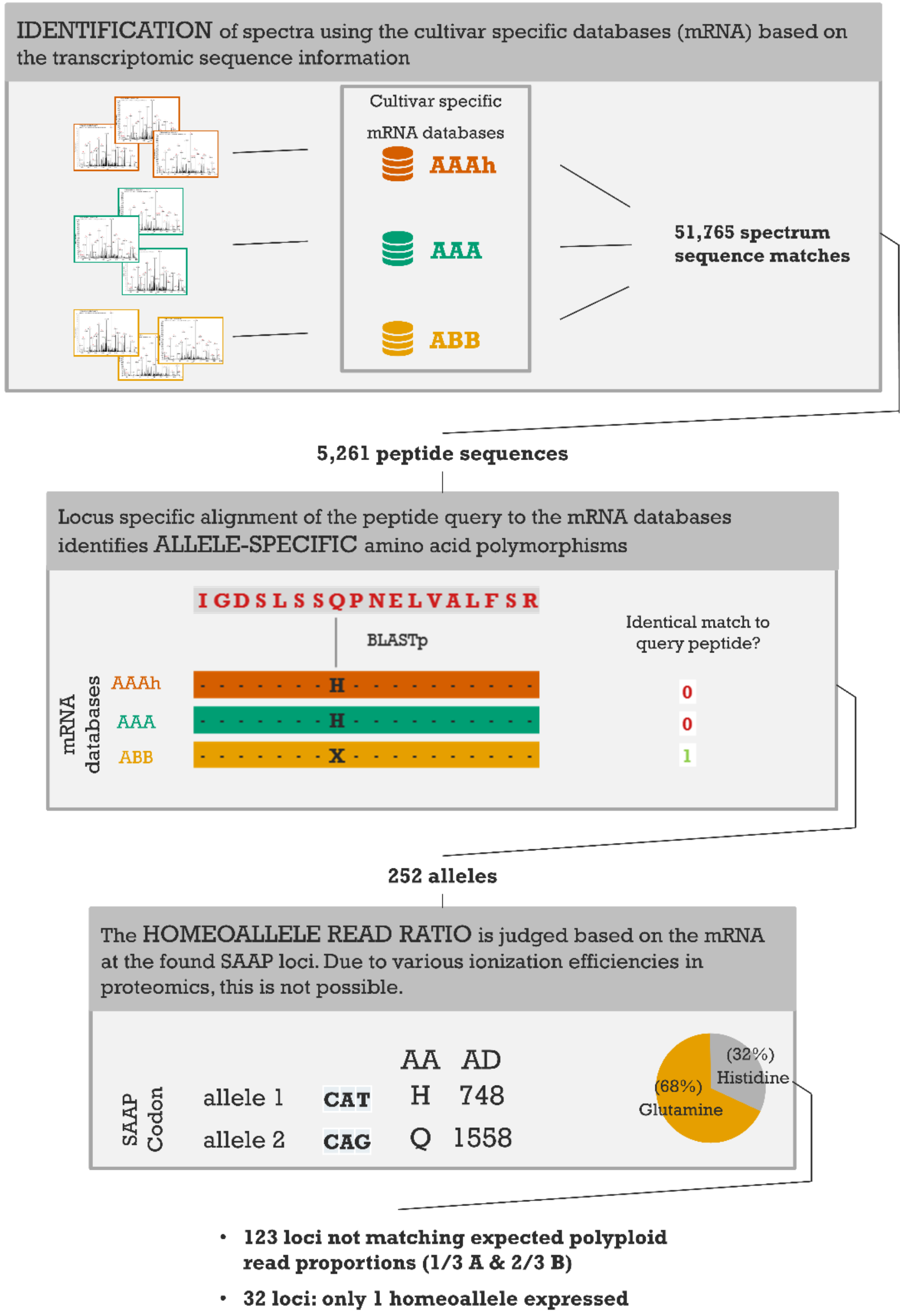

Supplementary Figure S4: Integration of transcriptomics and proteomics allows to pick up allele- specific protein sequences and quantify the different homeoalleles. Spectra are identified using cultivar specific databases derived from the transcriptomic sequence information. Allele-specific sequences are picked up by BLASTp alignment of any found peptide to three cultivar specific databases (mRNA seq based). Allele-specific databases (mRNA seq based). Allele-specific sequences match identical in one while non-identical in the other databases. In case of ambiguous amino acid in the database (X, caused by triploid nature of banana) the mRNA based vcf file (codon level) is assessed. The homeoalleles are quantified by their allele depth (AD) using the vcf-file (mRNA seq based) at the SNP location.
